# Metabolic Profiling and Antioxidant Assay of Metabolites from Three Radish Cultivars (*Raphanus sativus*)

**DOI:** 10.3390/molecules21020157

**Published:** 2016-01-28

**Authors:** Chang Ha Park, Thanislas Bastin Baskar, Soo-Yun Park, Sun-Ju Kim, Mariadhas Valan Arasu, Naif Abdullah Al-Dhabi, Jae Kwang Kim, Sang Un Park

**Affiliations:** 1 Department of Crop Science, Chungnam National University, 99 Daehak-Ro, Yuseong-gu, Daejeon 305-764, Korea; parkch804@gmail.com (C.H.P.); bastinbt20@yahoo.com (T.B.B.); 2 National Academy of Agricultural Science, Rural Development Administration, Wanju-gun, Jeollabuk-do 565-851, Korea; psy22@korea.kr; 3 Department of Bio-Environmental Chemistry, Chungnam National University, 99 Daehak-Ro, Yuseong-Gu, Daejeon 305-764, Korea; kimsunju@cnu.ac.kr; 4 Department of Botany and Microbiology, Addiriyah Chair for Environmental Studies, College of Science, King Saud University, P.O. Box 2455, Riyadh 11451, Saudi Arabia; mvalanarasu@gmail.com (M.V.A.); naldhabi@ksu.edu.sa (N.A.A.-D.); 5 Division of Life Sciences and Bio-Resource and Environmental Center, Incheon National University, Incheon 406-772, Korea

**Keywords:** radish, anthocyanin, phenolic, flavonoid, metabolic profiling, antioxidant assay

## Abstract

A total of 13 anthocyanins and 33 metabolites; including organic acids, phenolic acids, amino acids, organic compounds, sugar acids, sugar alcohols, and sugars, were profiled in three radish cultivars by using high-performance liquid chromatography (HPLC) and gas chromatography time-of-flight mass spectrometry (GC-TOFMS)-based metabolite profiling. Total phenolics and flavonoids and their *in vitro* antioxidant activities were assessed. Pelargonidins were found to be the major anthocyanin in the cultivars studied. The cultivar Man Tang Hong showed the highest level of anthocyanins (1.89 ± 0.07 mg/g), phenolics (0.0664 ± 0.0033 mg/g) and flavonoids (0.0096 ± 0.0004 mg/g). Here; the variation of secondary metabolites in the radishes is described, as well as their association with primary metabolites. The low-molecular-weight hydrophilic metabolite profiles were subjected to principal component analysis (PCA), hierarchical clustering analysis (HCA), Pearson’s correlation analysis. PCA fully distinguished the three radish cultivars tested. The polar metabolites were strongly correlated between metabolites that participate in the TCA cycle. The chemometrics results revealed that TCA cycle intermediates and free phenolic acids as well as anthocyanins were higher in the cultivar Man Tang Hong than in the others. Furthermore; superoxide radical scavenging activities and 1,1-diphenyl-2-picrylhydrazyl (DPPH) radical scavenging were investigated to elucidate the antioxidant activity of secondary metabolites in the cultivars. Man Tang Hong showed the highest superoxide radical scavenging activity (68.87%) at 1000 μg/mL, and DPPH activity (20.78%), followed by Seo Ho and then Hong Feng No. 1. The results demonstrate that GC-TOFMS-based metabolite profiling, integrated with chemometrics, is an applicable method for distinguishing phenotypic variation and determining biochemical reactions connecting primary and secondary metabolism. Therefore; this study might provide information on the relationship between primary and secondary metabolites and a synergistic antioxidant ability derived from the secondary metabolites in the radish cultivars.

## 1. Introduction

Radish (*Raphanus sativus* L.), a member of the Cruciferae family, is a root vegetable crop, cultivated and consumed around the world [1]. Radish cultivars are classified into two groups—spring/summer and winter cultivars—according to the season when the crops are cultivated. Cultivars in the first group are grown during the short spring and summer season. On the other hand, the winter cultivars are grown in autumn [2]. Previously, the aim of radish breeding was to select for improved abilities to adapt to various cultivation conditions and to improve pest resistance. In the past three decades, changing consumer preferences have led past breeding processes to be replaced with new breeding methods on the basis of morphological traits including shape, size, color, nutrition requirement, and cultivation requirements. Major efforts in breeding have enabled so-called red radish cultivars, which have improved functionality such as root shape, *i.e.*, globe-, ovula-, long-, flattened-, and pear-shape, and colors that range in variety from light to deep red, to appear on the market [3,4,5,6].

Red radish cultivars are a potential source of natural colorants due to the presence of anthocyanins, which have high stability and are highly similar to the artificial pigment, Food Red No. 40 [7,8,9]. Anthocyanins can be classified as malvidin, delphinidin, pelargonidin, cyanidin, petunidin, and peonidin derivatives according to the number of hydroxyl groups, the nature, position, and number of sugars present, as well as the nature and number of aliphatic or aromatic acid groups attached to the sugars [10]. Pelargonidin-, cyanidin-, and delphinidin-based anthocyanins are involved in color determination, *i.e.*, brick red/scarlet, red/magenta, and violet/blue color, respectively [11]. Interestingly, pelargonidin-based anthocyanins are mainly found in red radishes, which allow red-radish anthocyanin composition to be distinguished from that of other plants containing cyanidin- or delphinidin-based derivatives [12,13,14,15]. Additionally, anthocyanins have well-known health benefits, including the ability to scavenge free radicals, inhibit cancer and diabetes, prevent neuronal and cardiovascular diseases, and suppress inflammation [16,17,18,19,20].

Metabolic profiling involves systematic phenotyping analysis to obtain information on the quantitative low-molecular-weight metabolites present in a biological system in order to comprehensively describe a variety of biological phenomena in combination with genome-wide gene-expression arrays. This is accompanied by the measurement of a broad range of metabolites including amino acids, organic acids, sugars, TCA intermediates, bile acids, simple fatty acids, and oligopeptides [21,22]. Gas chromatography mass spectrometry (GC-MS) is a multidimensional analytical technique used for metabolic profiling. It has long been utilized in various fields of biological science and enables the identification and quantification of diverse metabolites in a single plant. Therefore, advances in this technology have led to the development of stable protocols for chromatography evaluation and interpretation [23,24,25,26]. In particular, the stable protocol GC-TOFMS, which has been developed for the analysis of small volatile molecules, has fast scan times that result in higher throughput (10–50 scans per second) and greater mass accuracy [23,27,28].

Free radicals are reactive oxygen species that are potentially toxic to cells. In humans, superoxide radicals are generated during metabolism under physiological conditions. These free radicals are scavenged or deactivated by antioxidants. Free radicals can damage lipids, proteins, and nucleic acids. Many enzymes function as antioxidants, including superoxide dismutase, catalase, and glutathione peroxidase, which are present in biological systems. Although non-enzymatic compounds are also used as antioxidant agents, including ascorbic acid, tocopherol, and β-carotene, which inhibit the free radical chain reactions. In plant biochemistry, 1,1-diphenyl-2-picrylhydrazyl (DPPH) is widely used in antioxidant assays and to evaluate the free radical scavenging properties of plant constituents [29].

The effect of oxidative stress in human beings has become a serious issue. The World Health Organization (WHO) reported that 80% of traditional medicines are obtained from bioactive compounds extracted from plants [30]. Antioxidant compounds are naturally present in plant sources and can be used medically through dietary supplementation to prevent oxidative stress, therefore preventing the need to take additional medication [31]. *Raphanus sativus* is an important root vegetable crop, which contains many medicinal and nutritional compounds [32]. Previous studies have reported that *Raphanus sativus* sprouts possess antioxidant activity [33], and these properties of polyphenolic compounds from this species have been investigated [34,35]. Radish leaves have also been reported to possess antioxidant activity [36]. Anthocyanin compounds have been shown to have antioxidant activity by the ORAC method [37].

Several studies have used HPLC to identify anthocyanins in red radish [38,39]. In particular, our previous published study reported that the presence of anthocyanins in peel and flesh of the three cultivars: Seo Ho, Man Tang Hong, and Hong Feng No. 1 [40]. However, a comprehensive interpretation of primary and secondary metabolites in red radish cultivars using HPLC analysis and GC-TOFMS-based metabolic profiling combined with chemometrics has not been published. Therefore, the aim of this study was to comprehensively describe the connection between diverse primary metabolites and secondary metabolites such as anthocyanins, total phenolics, and total flavonoids among the three cultivars. Additionally, we studied SOD-like activities, and DPPH radical scavenging was employed to identify the relationship between the antioxidant capacity and the natural products.

## 2. Results and Discussion

### 2.1. Anthocyanin Analysis

HPLC analysis revealed that pelargonidins were the major anthocyanidins in the radish cultivars, since among a total of 13 anthocyanins detected, 12 were pelargonidin-based, whereas one was a cyanidin-based anthocyanin derivative (Table 1).

**Table 1:** Anthocyanin content (mg/g dry wt.) in red radish.

No.^a^	RT (min)	Trivial Name	Seo Ho	Man Tang Hong	Hong Peng No. 1
1	8.073	Pelargonidin 3-diglucoside-5-glucoside	ND ^b^	0.08 ± 0.00	0.01 ± 0.00
2	8.966	Pelargonidin 3-diglucoside-5-(malonyl)glucoside	ND	0.06 ± 0.00	0.01 ± 0.00
3	10.846	Pelargonidin 3-(caffeoyl)diglucoside-5-glucoside	ND	0.02 ± 0.01	ND
4	12.172	Cyanidin 3-(glucosyl)rhamnoside	ND	0.09 ± 0.01	0.01 ± 0.00
5	12.994	Pelargonidin 3-(*p*-coumaroyl)diglucoside-5-(malonyl)glucoside	ND	0.16 ± 0.01	0.04 ± 0.00
6	13.309	Pelargonidin 3-(caffeoyl)diglucoside-5-(malonyl)glucoside	ND	0.02 ± 0.00	ND
7	14.668	Pelargonidin 3-(*p*-coumaroyl)diglucoside-5-(malonyl)glucoside	ND	0.22 ± 0.01	0.02 ± 0.00
8	15.424	Pelargonidin 3-(*p*-coumaroyl)(caffecoyl)diglucoside-5-(malonyl)glucoside	ND	0.19 ± 0.01	0.01 ± 0.00
9	15.954	Pelargonidin 3-(feruloyl)diglucoside-5-(malonyl)glucoside	ND	0.02 ± 0.00	ND
10	16.674	Pelargonidin 3-(*p*-coumaroyl)diglucoside-5-(malonyl)glucoside	ND	0.45 ± 0.02	0.10 ± 0.00
11	17.782	Pelargonidin 3-(feruloyl)diglucoside-5-(malonyl)glucoside	ND	0.47 ± 0.01	0.03 ± 0.00
12	20.916	Pelargonidin 3-(feruloyl)(caffecoyl)diglucoside-5-(malonyl)glucoside	ND	0.04 ± 0.01	ND
13	25.130	Pelargonidin 3-(*p*-coumaroyl)(feruloy)diglucoside-5-(malonyl)glucoside	ND	0.07 ± 0.01	ND
Total			ND	1.89 ± 0.07	0.23 ± 0.00

All the values in the table were expressed as Means ± Standard deviation (SD). The mean is an average of three samples obtained from the triplicated experiments. ^a^ No., the elution order of anthocyanins in HPLC analysis. ^b^ ND, not detected.

The cultivar Man Tang Hong contained the highest amount of total anthocyanin (1.89 ± 0.07 mg/g dry weight [wt.]), followed by Hong Feng No. 1 (0.23 ± 0.00 dry wt.). No anthocyanins were detected in the cultivar Seo Ho. In addition, Man Tang Hong contained the highest levels of all individual anthocyanins. Pelargonidin 3-(caffeoyl)-diglucoside-5-glucoside, 3-(caffeoyl)diglucoside-5-(malonyl)glucoside, and 3-(feruloyl)(caffecoyl)-diglucoside-5-(malonyl)glucoside were not detected in Hong Feng No. 1. These results concur with a previously published report on anthocyanin modifications and accumulation in these cultivars [40].

### 2.2. Total Phenolics and Flavonoid Contents

Total phenolics identified through the Folin-Denis assay showed that the cultivar ManTang Hong contained the highest amount (0.0664 ± 0.0033 mg/g). These levels were 4.53 times higher than in the cultivar Seo Ho (0.0147 ± 0.0010 mg/g) and 7.58 times higher than in the cultivar Hong Feng No. 1 (0.0088 ± 0.0006 mg/g). Similar data were obtained for the total flavonoid content. The highest accumulation of flavonoids was recorded in Man Tang Hong (0.0096 ± 0.0004 mg/g), followed by Seo Ho (0.0060 ± 0.0007 mg/g). On the other hand, Hong Feng No. 1 did not contain any flavonoids (Table 2).

**Table 2:** Total polyphenolics and flavonoid contents of ethanol extract from *Raphanus sativus*.

Cultivars	Total Phenolics (mg/g)	Total Flavonoid (mg/g)
Seo Ho	0.0147 ± 0.0010 ^b^	0.0060 ± 0.0007 ^b^
Man Tang Hong	0.0664 ± 0.0033 ^a^	0.0096 ± 0.0004 ^a^
Hong Feng No. 1	0.0088 ± 0.0006 ^b^	0 ^c^

All the values in the table were expressed as means ± standard deviation (SD). The mean is an average of three samples obtained from the triplicated experiments. Mean values with a different superscripted letter (a, b, and c, respectively) were significantly different at *p* < 0.05 using the Duncan Multiple Range Test (DMRT).

### 2.3. Metabolic Profiles among Radish Cultivars Using GC-TOFMS Analysis

**Figure 1:**
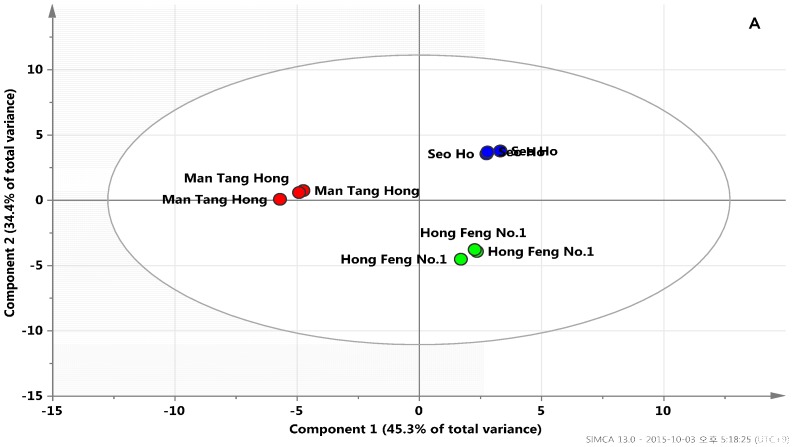

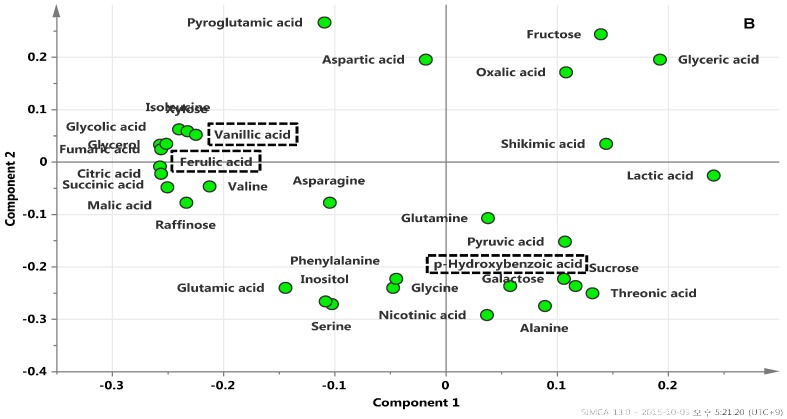
Scores (**A**) and loading plots (**B**) of principal components 1 and 2 of the PCA results obtained from polar metabolite data on different radish cultivars. Secondary metabolite was marked by a dotted box.

Metabolic profiling technology enables the rapid and precise assessment of metabolites and the subsequent metabolic pattern recognition of biological samples. Therefore, here, GC-TOFMS was used to identify and quantify the low-molecular-weight hydrophilic compounds present in three radish samples. After performing data processing and peak determination as described above, a total of 33 metabolites were detected in the radish samples. PCA has been the most frequently used as a clustering technique to determine how one sample is distinct from another, which metabolites contribute most to this difference, and whether these metabolites are correlated. Quantification data of 33 metabolites normalized to the IS signal intensity were subjected to PCA to investigate the data structure. Among these radish cultivars, PCA with the first two components explaining 79.7% of the total variance of the samples showed clear differentiation. Variance of the genotype differences between Mang Tang Hong and the other cultivars was successfully captured horizontally in principal component (PC). Additionally, the metabolomes between Hong Feng No. 1 and Seo Ho were separated by PC2 vertically. The identification of compounds revealed that the prominent variance within a population and the determination of closely related compounds is possible using PCA [41]. To further investigate contributors to the components, the metabolic loadings in PC1 and PC2 were compared. In PC1, the corresponding loading was negative for citric acid, succinic acid, and fumaric acid (Figure 1).

**Figure 2:**
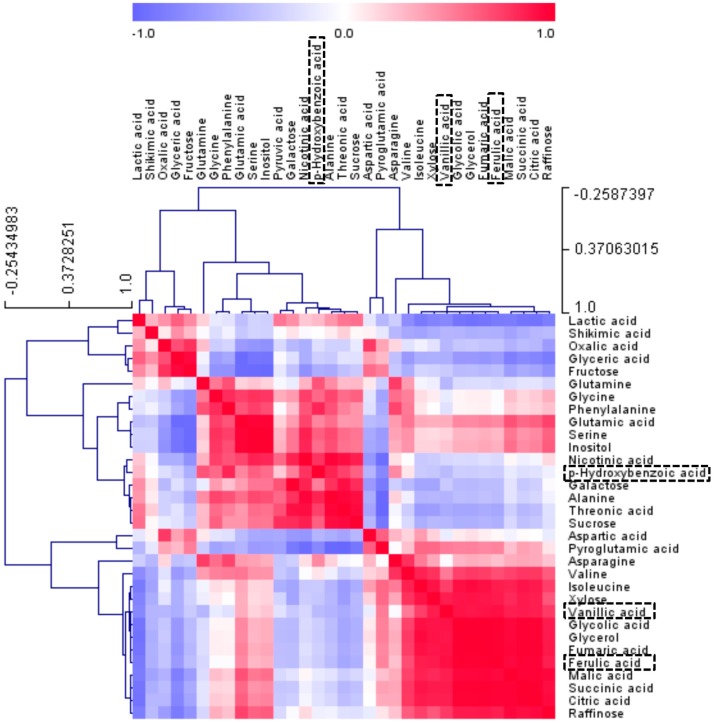
Correlation matrix of metabolites from three radish cultivars. Each square indicates the Pearson’s correlation coefficient of a pair of compounds, and the value of the correlation coefficient is represented by the intensity of the blue or red color, as indicated on the color scale. Secondary metabolite was marked by a dotted box.

In Figure 2, the relationship between the levels of 33 metabolites present in the radishes was investigated through Pearson’s correlation analysis and HCA of the accessions. The most distinct aspect was found in TCA cycle intermediates, which revealed significant positive correlations among each other. The result complies with the findings from PCA loading plots (Figure 1), indicating that PCA can be used to visualize complex data. Citric acid contents were positively correlated with the branched organic acids, succinic acid (*r* = 0.9962, *p* < 0.0001), fumaric acid (*r* = 0.9847, *p* < 0.0001), and malic acid (*r* = 0.9748, *p* < 0.0001), respectively. Likewise, the relationship between ferulic acid and vanillic acid (*r* = 0.9172, *p* < 0.0005), which are precursors for the anthocyanin biosynthesis pathway, was significantly positive 

### 2.4. In-Vitro Antioxidant Assay

#### 2.4.1. Superoxide Radical Scavenging Activity

**Figure 3:**
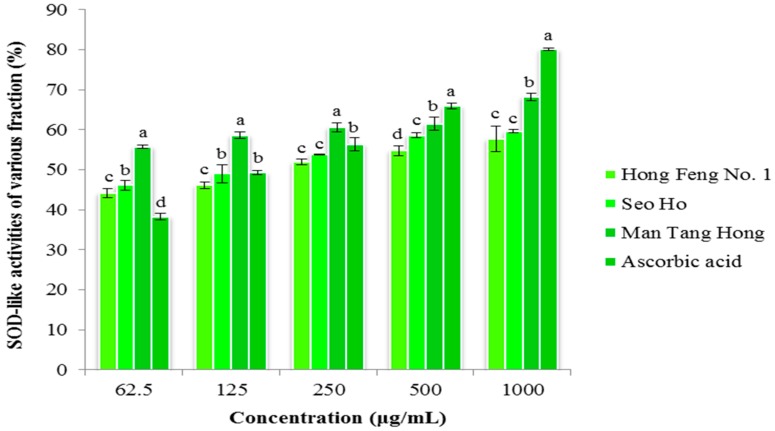
Superoxide Radical Scavenging activity of various fractions from *Raphanus sativus.* All the values in the figure are expressed as means (%) of triplicated experiments ± SD of three experiments. Mean values in a different letter (a, b, c and d, respectively) were significantly different at *p* < 0.05 using Duncan Multiple Range Test (DMRT).

Superoxide radical scavenging activity was determined using compounds from three cultivars and the percentage inhibition of superoxide radical generation was measured (Figure 3). The cultivar Man Tang Hong had the highest superoxide radical scavenging activity (68.87% in 1000 μg/mL), followed by Seo Ho (59.62% in 1000 μg/mL), and then Hong Feng No. 1 (57.69% in 1000 μg/mL). Furthermore, Man Tang Hong reached 50% inhibition activity at the initial concentration of compound used (62.5 μg/mL) and this was reached by the other two cultivars at 250 μg/mL.

#### 2.4.2. DPPH Assay

**Figure 4:**
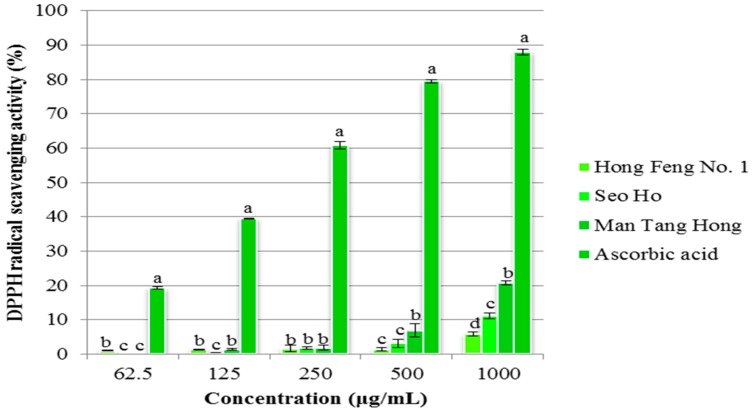
Superoxide Radical Scavenging activity of various fractions from *Raphanus sativus.* All the values in the figure are expressed as means (%) of triplicated experiments ± SD of three experiments. Mean values in a different letter (a, b, c and d, respectively) were significantly different at *p* < 0.05 using DMRT.

DPPH radical scavenging activity is one of the most efficient methods used to screen the antioxidant activity of plant extracts. DPPH free radical scavenging activities of compounds extracted from three different cultivars, Seo Ho, Man Tang Hong, and Hong Feng No. 1, are shown in Figure 4. The compounds from these cultivars had concentration-dependent scavenging activity. The cultivar Man Tang Hong, revealed the highest DPPH radical scavenging activity (20.78% in 1000 μg/mL), followed by Seo Ho (11.08% in 1000 μg/mL), and Hong Feng No. 1 (5.84% in 1000 μg/mL).

*Raphanus sativus* is one of the most widely studied root vegetable crops worldwide. Phytochemicals, polyphenols, and flavonoids are essential nutrients that are found in cruciferous vegetables such as broccoli, cabbage, kale, cauliflower, and radish, which have an inhibitory affect against cardiovascular diseases and cancers as shown by epidemiological data [42,43,44,45,46]. Different organs of radish, including the roots, seeds, and leaves, contain compounds that have different medicinal properties, Asthma and other respiratory complaints have been successfully treated with radish seeds. There are numerous types of anthocyanins, which are differentiated according to the type and number of conjugated sugars, number, and position of the hydroxyl and methoxyl groups as substituent on the B ring, and the presence or absence of an acyl group. The six most significant classifications are pelargonidin (Pg), cyanidin (Cy), peonidin (Pn), delphinidin (Dp), petunidin (Pt), and malvidin (Mv) [47]. Phenolic compounds (flavonols and anthocyanins), carotenoids, vitamins, and minerals are also found in high levels in *Brassicaceae* foods [48]. In the present study, we aimed to identify anthocyanin, phenolic, and flavonoid compounds present in three radish cultivars. Previous studies have reported only four types of pelargonidin identified in this species during fermentation [49]. We identified 12 types of pelargonidin present in radish. About 0.47 ± 0.01 (mg/g dry wt.) content was found in the Mang Tang Hong cultivar and was very low in the other two cultivars. The total phenolic content from this radish plant has been previously reported [35,50]. Phenolic compound synthesis was affected by many factors such as plant breeding, ontogenetic stage, post-harvest, handling, and biotic and abiotic factors [51]. 

Some researchers have noted that the characterization of anthocyanins in radish is dependent on the variety studied [52]. The quantification of anthocyanins including pelargonidin, cyanidin, and delphinidin was investigated by Hanlon and Barnes [4], and many research groups [12,53] also found that the major anthocyanins in radish sprouts are acylated pelargonidins, such as in the Daikon cultivar [54], while other researchers showed that cyanidin-based pigments could be isolated from red radish (*R. sativus* L. var. Benikanmi) [55], radish cultivar Sango sprouts [56], and Purple Bordeaux radish [57]. However, other studies have shown that glucoraphasatin purified from radish sprouts has some antioxidant capacity [58], which contributed to the total antioxidant capacity of radish sprout extract [59]. Phenolic compounds in plants are commonly synthesized through the phenylpropanoid pathway. Cruciferae (Brassicaceae) are prominent root vegetables and their utilization decreases the risk of developing various types of cancer [44,60]. Several studies have reported that the composition of phenolic contents used to distinguish plants could result from multiple factors, including the methodology (extraction procedure, different susceptibilities to degradation, type of chromatography and quantification), growth, plant species, and storage environments [61,62,63]. Thus, many research groups have focused on discovering the bioactive compounds from this radish plant that might be promising chemopreventive agents. In this study, we report that the highest levels of phenolic compounds are found in the cultivar Mang Tang Hong compared to other two cultivars. Accordingly, the flavonoid content was the same in the Mang Tang Hong cultivar.

In these results, metabolites that involved in closely related pathways revealed a high correlation, demonstrating that this is a powerful tool for tracking metabolic links. The results of the correlation and PCA analyses showed that the TCA cycle intermediates and phenolic acids were found at relatively higher levels in Mang Tang Hong than in the other cultivars. Flavonol biosynthesis and cellular catabolism associated with intermediates of the TCA cycle were reported in a previous study [64]. 2-Oxoglutarate (2-OG), a key organic acid of the TCA cycle [65,66], is also an obligatory substrate for 2-OG-dependent dioxygenases (2-ODDs). In flavonoid biosynthesis, four types of 2-ODDs; flavonol synthase (FLS), flavanone 3-hydroxylase (F3H), anthocyanin synthase, and flavone synthase I (FS-I), have been characterized as key enzymes in late steps of flavonoid aglycone formation resulting in species specific flavonoid profiles [67,68,69].

The antioxidant activity of plant extracts depends on the levels of phenolic, flavonoid, and anthocyanin compounds [70]. Flavonoid compounds contain more hydroxyl groups and commonly possess high antioxidant activities [71]. Previous studies confirmed the antioxidant activity of radish extract by the ORAC method [72]. Red radish was found to have higher antioxidant activity than previously reported [73]. In this study, we report antioxidant activity using two methods, DPPH radical scavenging and superoxide radical scavenging activity, which resulted in higher scavenging activity being found in extracts from Mang Tang Hong compared to other two cultivars. The scavenging activity increased depending on the concentration of the compound used, and is comparable with the standard ascorbic acid. Crude radish extract is also known to possess antioxidant enzyme activity and the antioxidant l-tryptophan has previously been reported to be produced from radish extracts [74]. 

## 3. Experimental Section 

### 3.1. Plant Materials 

Three red radish cultivars (Seo Ho, Man Tang Hong, and Hong Feng No. 1) were cultivated in a greenhouse at the experimental farm of the Rural Development Administration (RDA, Suwon, Korea) in 2009. The cultivars were harvested at maturity (14–18 weeks). Next, each sample was freeze-dried twice at −80 °C for 48 h and then ground into powder. Nitroblue tetrazolium (NBT), hydroxylamine hydrochloride, ascorbic acid, and DPPH were purchased from Sigma-Aldrich, St. Louis, MO, USA).

### 3.2. Anthocyanin Extraction and Analysis

Powder (100 mg) ground from each of the three freeze-dried radish cultivars, was placed in a 2-mL Eppendorf tube. Extraction solution (water/formic acid, 95:5, *v*/*v*, 2 mL) was added to the tube, the sample was then vortexed for 5 min and sonicated for 20 min. Next, the supernatant obtained through centrifugation at 8000 rpm for 15 min was filtered through a through a 0.45-μm PTFE hydrophilic syringe filter (Advantec DISMIC-13HP, ToyoRoshi Kaisha, Ltd., Tokyo, Japan), prior to HPLC injection. Anthocyanin HPLC analysis was carried out on a Flexar HPLC system (Perkin-Elmer, Shelton, CT, USA) coupled to a PDA LC detector. The separation of individual anthocyanins was achieved on a Synergy 4 μ Polar-RP 80A (250 × 4.6 mm, i.d.) column with a Security Guard Cartridges Kit (AQ C18, 4 × 3 mm, i.d.; Phenomenex, Torrance, CA, USA) using the mobile phase solvents composed of eluent (A) water/formic acid (95:5, *v*/*v*) and eluent (B) acetonitrile/formic acid (95:5, *v*/*v*) at a flow rate of 1 mL/min. The column and guard column in the oven were thermostatically controlled at 40 °C. The gradient program, modified from previous studies [75,76], was set as follows: 0–2 min, 5%–18% B; 2–4 min, 18% B; 4–9 min, 18%–20% B; 9–14 min, 20% B; 14–19 min, 20%–21% B; 19–24 min, 21% B; 24–24.1 min, 21%–5% B; and 24.1–30 min, 5% B (total 30 min). The injection volume and detection wavelength were 10 μL and 520 nm, respectively. The procedure was repeated in triplicate.

### 3.3. GC-TOFMS Analysis of Polar Metabolites

Polar metabolites were extracted as described previously [41]. Metabolites were extracted from the powdered sample (10 mg) by adding 1 mL 2.5:1:1 (*v*/*v*/*v*) methanol:water:chloroform. Ribitol (60 μL, 0.2 mg/mL) was added an as internal standard (IS). For GC-TOFMS analysis, a 2-stage chemical derivatization (oximation and trimethylsilyl etherification) was performed on the extracted metabolites. After derivatization, GC-TOFMS procedures were performed, according to a method previously reported [41]. ChromaTOF software was used to support peak findings prior to quantitative analysis and for automated deconvolution of reference mass spectra. The NIST and in-house libraries for standard chemicals were utilized to identify the compounds. Quantitative calculations used to determine the concentrations of all analytes were based on the peak area ratios for each relative to the peak area of the IS.

### 3.4. In-Vitro Antioxidant Assay

#### 3.4.1. Superoxide Radical Scavenging Activity

Superoxide scavenging activity of analyzed compounds from three different cultivars, Seo Ho, Man Tang Hong, and Hong Feng No. 1, was measured using NBT according to the method described by Sunil *et al.* [77]. Nitrate reduction occurs during the generation of superoxide radicals by oxidation of hydroxylamine hydrochloride in the presence of NBT. Test solutions of the compound (62.5–1000 μg/mL) were placed in a test tube. To this, sodium carbonate (1 mL, 50 mM), NBT (0.4 mL, 24 mM), and EDTA (0.2 mL, 0.1 mM) solutions were added. Hydroxylamine hydrochloride (about 0.4 mL, 1 mM) was added to initiate the reaction; the reaction mixture was then incubated at 25 °C for 15 min and reduction of NBT was measured at 560 nm in a UV spectrophotometer. A sample not containing extract was used as a control and ascorbic acid was used as a standard. Superoxide radical scavenging activity (%) = [(*A0 −* *A1/A0)* × 100], where *A0* is the absorbance of the control at 15 min and *A1* is the absorbance of the sample at 15 min. All samples were analyzed in triplicate.

#### 3.4.2. DPPH Assay

DPPH scavenging activity of compounds from three different cultivars, Seo Ho, Man Tang Hong, and Hong Feng No. 1, was evaluated as previously described [78]. First, we prepared 0.15% DPPH in ice cold methanol. The reaction mixture contained methanol (3.8 mL) to which various concentrations of extracts (62.5–1000 μg/mL) and DPPH solution (200 μL) were added. Samples were then incubated at room temperature for 30 min in the dark. After incubation, the absorbance was read at 517 nm using a UV spectrophotometer. The DPPH radical scavenging activity was calculated, and vitamin C was used as a standard. DPPH radical scavenging activity was calculated as (%) [(*A0 −* *A1/A0*) × 100], where *A0* is the absorbance of the control at 30 min and *A1* is the absorbance of the sample at 30 min. All samples were analyzed in triplicate.

### 3.5. Statistical Analysis

The computer software Statistical Analysis System (SAS) 9.4 (2013; SAS Institute, Inc., Cary, NC, USA) was used for statistical analyses. Each mean was separated to determine significant differences by Duncan’s Multiple Range Test (DMRT). All data are presented as mean ± standard deviation of triplicate experiments. The relative quantification data acquired from GC-TOFMS were subjected to PCA (SIMCA-P version 13.0; Umetrics, Umeå, Sweden) to evaluate the relationships in terms of similarity or dissimilarity between groups of multivariate data. The PCA output consisted of score plots used to visualize the contrast between different samples, and loading plots to explain the cluster separation. The data file was scaled with unit variance scaling before all the variables were subjected to PCA. Pearson’s correlation analysis was performed using the SAS 9.2 software package. Correlation analysis was performed among the relative levels of 33 metabolites using a standardization procedure. HCA and heat map visualization of the correlation coefficients were performed using the software Multi Experiment Viewer version 4.4.0 (Dana-Farber Cancer Institute, Boston, MA, USA, http://www.tm4.org/mev/).

## 4. Conclusions 

In this study, we report a connection between the primary and secondary metabolites produced from the radish plants. Among the three cultivars, Man Tang Hong showed the highest concentrations of primary metabolites such as TCA cycle intermediates and phenolic acids. This might allow the cultivar to synthesize more secondary metabolites, including total phenolics, flavonoids and anthocyanins. Subsequently, their highest levels of secondary metabolites explained the most scavenging activity of the cultivar. Additionally, the results in the present study confirm that HPLC and GC-TOFMS-based metabolite profiling, combined with chemometrics, are powerful tools to determine phenotypic variation and identify metabolic links between primary and secondary metabolism. 
